# Intramural Comparison of NIST Laser and Optical Fiber Power Calibrations

**DOI:** 10.6028/jres.109.019

**Published:** 2004-04-01

**Authors:** John H. Lehman, Igor Vayshenker, David J. Livigni, Joshua Hadler

**Affiliations:** National Institute of Standards and Technology, Boulder, CO 80305

**Keywords:** absolute responsivity, calorimeter, cryogenic radiometer, intercomparison, laser, optical power, optical fiber, pyroelectric detector, spectral responsivity

## Abstract

The responsivity of two optical detectors was determined by the method of direct substitution in four different NIST measurement facilities. The measurements were intended to demonstrate the determination of absolute responsivity as provided by NIST calibration services at laser and optical-communication wavelengths; nominally 633 nm, 850 nm, 1060 nm, 1310 nm, and 1550 nm. The optical detectors have been designated as checks standards for the purpose of routine intramural comparison of our calibration services and to meet requirements of the NIST quality system, based on ISO 17025. The check standards are two optical-trap detectors, one based on silicon and the other on indium gallium arsenide photodiodes. The four measurement services are based on: (1) the laser optimized cryogenic radiometer (LOCR) and free field collimated laser light; (2) the C-series isoperibol calorimeter and free-field collimated laser light; (3) the electrically calibrated pyroelectric radiometer and fiber-coupled laser light; (4) the pyroelectric wedge trap detector, which measures light from a lamp source and monochromator. The results indicate that the responsivity of the check standards, as determined independently using the four services, agree to within the published expanded uncertainty ranging from approximately 0.02 % to 1.24 %.

## 1. Introduction

The responsivity of a single optical detector determined from independent comparisons is a means of assuring that our stated uncertainties for a given measurement service are reasonable. Furthermore, such comparisons are a means of complying with the ISO 1725 quality system, the acceptance of which has been agreed upon by the world’s National Measurement Institutes [1]. Through these intercomparisons, we have become a customer of our own services and are able to rigorously evaluate our performance.

We presently have four measurement systems for measuring continuous-wave (CW) laser power at relatively low power levels (milliwatts and less). The oldest among the services was established in the late 1960s and is based on an isoperibol calorimeter, which we call the C-series calorimeter. This measurement device is electrically calibrated and is traceable to electrical standards (the volt and ohm). Since the C-series calorimeter was developed and as the demand for higher accuracy continues, we have more recently developed a measurement service based on a cryogenic radiometer as a primary standard; the Laser Optimized Cryogenic Radiometer (LOCR). To meet growing customer demand for routine calibration of laser and optical-fiber power meters (OFPMs), we have developed two additional calibration services based on comparisons with pyroelectric detectors for absolute responsivity of fiber-coupled power meters and relative spectral responsivity. Among these four calibration services, absolute responsivity of fiber-coupled power meters, or OFPMs, at common telecommunications wavelengths (for example, 850 nm, 1310 nm, and 1550 nm) is the most frequently requested. The demand for OFPM calibrations is approximately 75 calibrations per year and continues to increase.

**Fig. 1:**
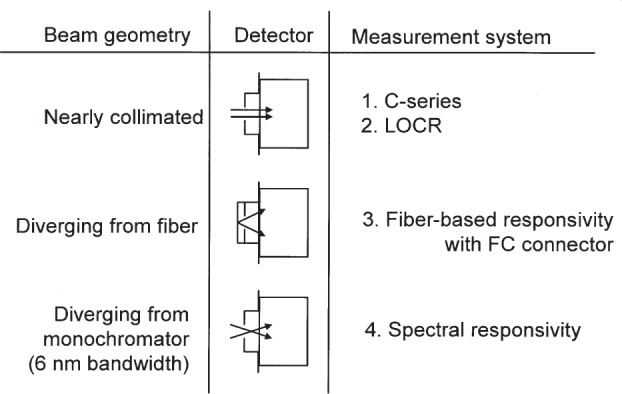
Relationship of the beam geometry and the detector input among the four measurement systems.

For the intramural comparison we used transfer standards capable of low measurement uncertainty [2,3]. These transfer standards are intended to have very high coupling efficiency, so that they may be used with the four measurement systems having various input-beam geometries, as shown in Fig. 1. These input geometries are: (1) free-field, nearly collimated laser light input; (2) laser light transmitted by single-mode fibers coupled with FC-type fiber connectors; (3) moderately diverging light from a lamp and monochromator. Where possible, we sought to repeat the responsivity measurements with the three laser-based measurement systems, using laser sources with nearly the same wavelengths. Nominally these wavelengths are: 514 nm, 633 nm, 850 nm, 1064 nm, 1310 nm, and 1550 nm. The spectral responsivity measurement system is capable of wavelength adjustment precision of about ±0.1 nm to approximate the wavelength of any of the laser sources, but the bandwidth is approximately 6 nm [4].

The results of this intramural comparison are given in several subject areas: a description of the two transfer standards, description of the four measurement systems with a statement of the measurement uncertainty, and a summary of results. The uncertainty is given with coverage factor, *k* = 2, in every case. The coverage factor corresponds to a level of confidence for the relative expanded uncertainty that is approximately 95 % [5].

## 2. Transfer Standards

The transfer standards, or check standards, for this comparison are photodiode-based optical detectors designed and built at NIST [2,3]. For convenience, we use the colloquial term “device under test,” or DUT, to identify these detectors. The optical configuration of each DUT is based on an optical trap having two photodiodes and a spherical mirror. This basic design has been employed in the past using three diodes (and no mirror) [6]. The presence of the spherical mirror reduces the external quantum efficiency of the trap (compared with the three-diode design), but increases the coupling efficiency for larger values of numerical aperture (NA) [3]. The trap based on silicon (Si) photodiodes is suitable for measurements requiring an NA as large as 0.26. The trap based on indium gallium arsenide (InGaAs) photodiodes is suitable for a slightly lower NA because of the diode packaging constraints (the size of the chip carrier), and the choice of the spherical mirror having a larger radius of curvature. DUT1 designates the detector based on Si photodiodes and DUT2 designates the detector based on InGaAs photodiodes.

We evaluated the detector responsivity in units of amperes per watt (A/W). The current generated by each DUT was measured by a commercially available picoammeter.

## 3. C-series Calorimeter

The C-series calorimeter is an isoperibol calorimeter first developed at the National Bureau of Standards around 1968 [7]. In principle, the measurement device and data analysis have changed very little since then. The calorimeter is considered to be isoperibol when the ambient temperature is constant while the calorimeter optical cavity temperature changes with time. The amount of optical power measured by the calorimeter is derived from knowledge of the exponential decrease in temperature of the calorimeter optical cavity, following a laser injection of optical energy for a known duration [8]. The calorimeter is electrically calibrated by substituting the laser injection with electrical heating, which may be measured accurately. The measurement procedure for the two check standards was identical, except for the laser wavelength used for each calibration.

**Fig. 2:**
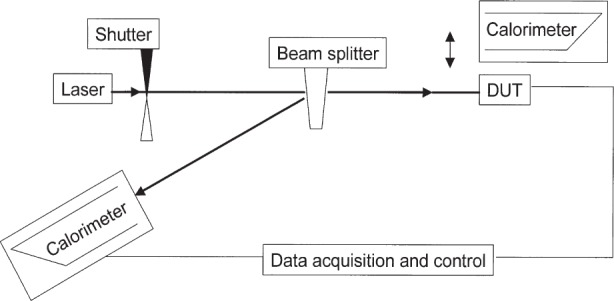
Measurement scheme for C-series calorimeter measurements.

The test procedure employs a beam splitter that reflects a portion of the laser source to a reference calorimeter and transmits a portion to a primary calorimeter as shown in Fig. 2. From this, the splitter ratio may be determined, which is simply the ratio of optical power in the transmitted beam to the reflected beam. With knowledge of the splitter ratio and the amount of optical power incident on the primary calorimeter, the responsivity of the DUT is determined.

The DUTs were compared to two NIST standard C-series calorimeters, designated C4-1 and C4-4, at three wavelengths: 632.8 nm (HeNe laser), 859.4 nm (diode laser), and 1064 nm (Nd:YAG laser), by use of the measurement scheme shown in Fig. 2. The laser beam had a diameter of 2 mm or less, and was centered on the DUT input aperture. The power impinging upon the test instrument was measured concurrently by means of the calibrated beam splitter and the NIST reference calorimeter. The splitter ratio of the calibrated beam splitter was determined before and after each detector calibration using the two standard calorimeters.

The uncertainty analysis of this measurement is given in detail elsewhere [9]. The expanded uncertainty (with a coverage factor of *k* = 2) of calibrations based on the primary standard typically ranges from approximately 0.5 % to 1 %. This value varies largely as a result of measurement noise due to laser power instability, which depends on the laser wavelength and power level.

## 4. Laser Optimized Cryogenic Radiometer

The NIST LOCR is based on a commercially available cryogenic radiometer [10], which relies on a servo control system to maintain a constant temperature during laser heating of the radiometer cavity. The electrical heating compensation (decrease in heating power), is equal to the amount of optical power absorbed by the radiometer cavity [11].

**Fig. 3:**
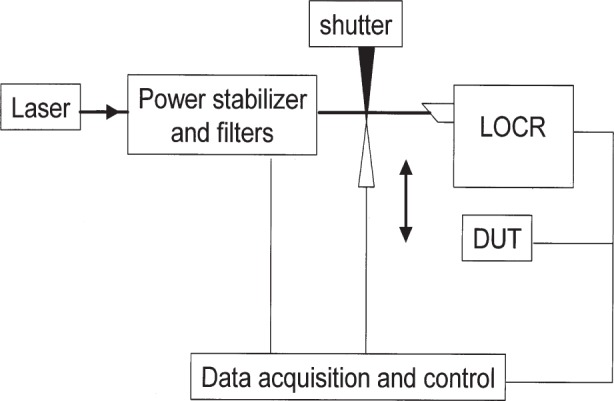
Measurement scheme for the LOCR-based calibrations.

The responsivities of DUT1 and DUT2 were determined by direct substitution of the LOCR using the calibration system shown in Fig. 3. Each DUT was in turn calibrated with a nominal power level of 1 mW. The calibration system used a laser power stabilizer and a spatial filter to remove scattered light. The optical power applied to the DUT was calculated by interpolating between power measurements performed with the LOCR before and after the test detector measurement, and then applying the appropriate correction factors.

Four correction factors were used: the LOCR window transmittance (TW), the LOCR receiver absorptance (AR), the relative aperture transmittance (TA), and the LOCR electrical calibration (kL). The detector’s absolute responsivity (R) in A/W is given by the equation:

$$R=\frac{O_{M}T_{W}A_{R}}{P_{S}T_{A}k_{L}},$$ (1)

where *P*_S_ is the applied power in watts, interpolated from bracketing primary standard measurements, and *O*_M_ is the detector output in amps.

The calibrations were performed using four laser sources with vacuum wavelengths of 514.6744 ± 0.0044 nm, 632.9918 ± 0.0054 nm, 1064.4209 ± 0.0054 nm, and 1550.4142 ± 0.0055 nm (all uncertainties *k* = 2). The laser radiation was contained in a single spectral line having an approximately Gaussian intensity profile with known 1/*e*^2^ diameter at the detector’s entrance aperture.

The uncertainty analysis of this measurement is given in detail elsewhere [11]. The expanded uncertainty (with a coverage factor of *k* = 2) of calibrations based on the primary standard typically ranges from approximately 0.02 % to 0.12 %.

## 5. Spectral Responsivity

**Fig. 4:**
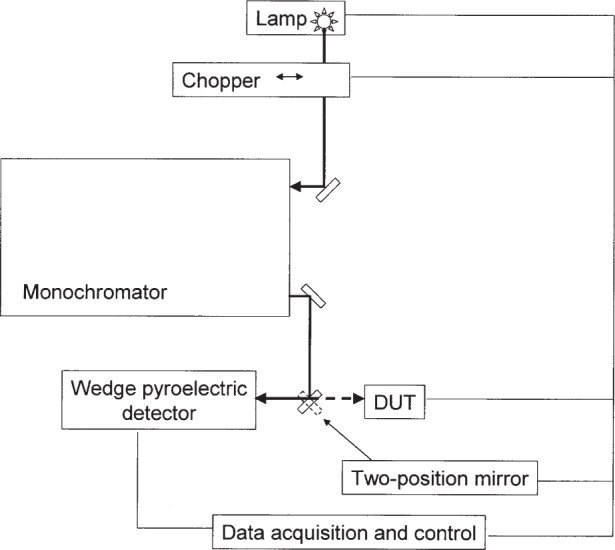
Measurement scheme for spectral responsivity.

The spectral responsivity of the DUT was determined by direct substitution with a temperature-stabilized wedge-trap pyroelectric detector [11]. The operation of the pyroelectric element is based on the volume average of the change in temperature with respect to time [12]. The wedge-trap is a secondary standard, with traceability to the C-series calorimeter at several laser wavelengths and a known value for the reflectance of the detector coating over a range of wavelengths. The spectral responsivity of each DUT was measured using the system shown schematically in Fig. 4. The measurement system is designed to accommodate a variety of commercial instruments that cover the spectrum of wavelengths ranging from 400 nm to 1800 nm.

A tunable monochromatic light source composed of a filament lamp, a grating monochromator, and a set of bandpass filters was used to calibrate the DUT. The output beam from the monochromator (transmitted through air) was directed alternately onto the DUT and the NIST transfer standard with a two-position mirror. The beam was focused (f# ≅ f/4) to a diameter of approximately 2 mm at the position of, and normal to, the plane of the test meter. The bandpass of the monochromator was less than 6 nm. The typical uncertainty value of this measurement is 1.24 % (*k* = 2) and the uncertainty analysis is given in detail elsewhere [4]. The largest contribution to the uncertainty is calibration of the transfer standard with the primary standard, stray light, and the fact that the bandwidth is as large as 6 nm.

## 6. Fiber-Coupled Absolute Responsivity

The absolute responsivity of each DUT was determined by direct substitution of the DUT and an electrically calibrated pyroelectric radiometer (ECPR). The amount of optical power measured by the ECPR is based on electrical substitution. The ECPR is considered a secondary standard traceable to the LOCR. In addition to being used to quantify the optical power absorbed by the ECPR, the electrical substitution provides thermal compensation for the pyroelectric response. The electrical substitution method is accomplished by heating the detector with an amount of electrical power that is equal to, and 180° out of phase with, the optical beam transmitted through the chopper.

**Fig. 5:**
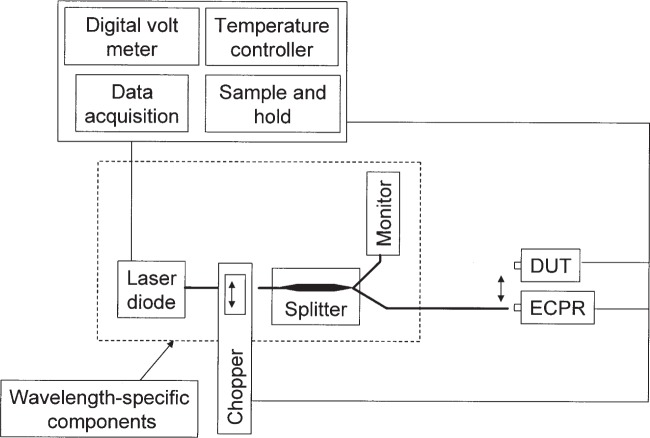
Diagram of the important components of the fiber-based measurement system. Note that when the fiber is connected to the ECPR the chopper is in the beam path, but the chopper is removed when the fiber is connected to the DUT.

Figure 5 shows the measurement system configuration for calibration of optical fiber power meters (OFPMs). The fiber-based measurement system is based on light emitted from a variety of fiber-coupled laser diodes at wavelengths 672.4 nm, 851.5 nm, 1306.5 nm and 1549.6 nm. Each laser source contains a laser diode whose output is transmitted through a fiber to a fiber splitter, from which about 1 % of the energy travels to a monitor detector. All system optical fibers are single mode. The remaining 99 % of the energy is transmitted through another fiber to the DUT. All the lasers (except for 1550 nm) are Fabry-Perot types and have several longitudinal spectral modes. The coherence length of each of these lasers is approximately a few centimeters. The 1550 nm laser is a distributed-feedback (DFB) laser with a coherence length of a few hundred meters.

A collimation fixture (not shown in Fig. 5) forms a gap in the fiber between the diode laser and the splitter. This fixture contains two lenses; one to collimate, the other to collect and focus light that is transmitted a short distance (free field) through the ECPR chopper wheel. When the chopper wheel is inserted into the gap, a chopped beam is then incident on the detectors (that is, the monitor and the ECPR). The chopper wheel is inserted into the collimation fixture gap each time the ECPR is used for measurements in this system but is removed when not using the ECPR.

The uncertainty value of this measurement is 0.4 % (*k* = 2) and the uncertainty analysis is given in detail elsewhere [13]. The largest contribution to the uncertainty is calibration with the primary standard.

## 7. Results

**Fig. 6:**
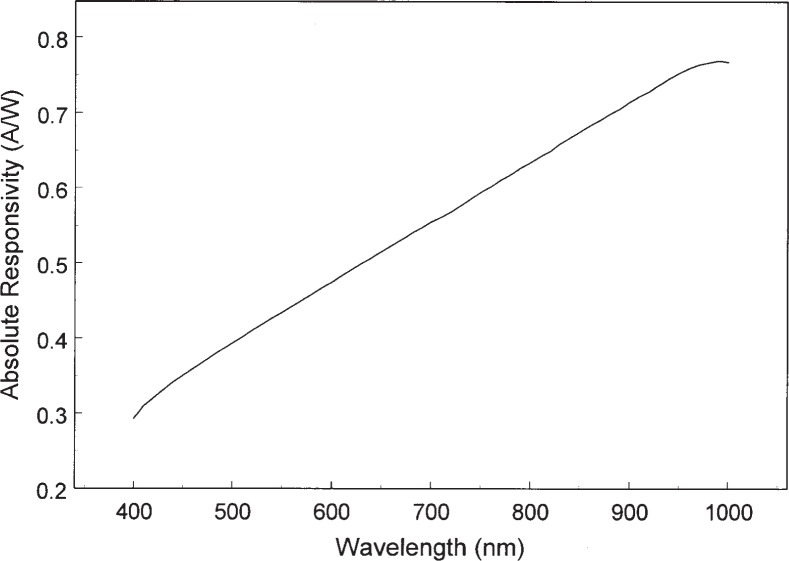
Absolute spectral responsivity of DUT1, the Si photodiode-based trap detector.

**Fig. 7:**
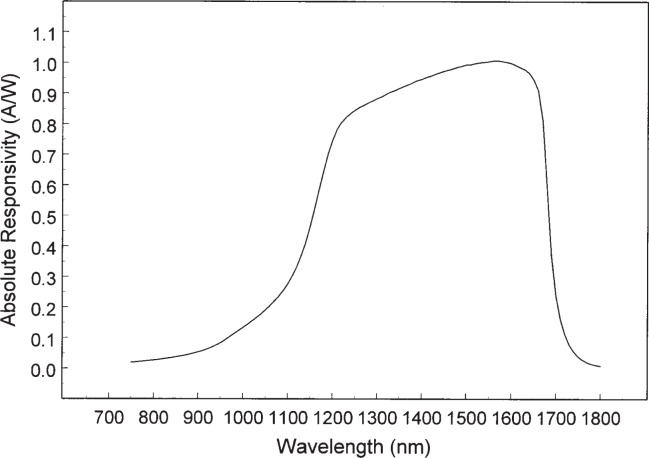
Absolute spectral responsivity of DUT2, the InGaAs photodiode-based trap detector.

The results are summarized two ways. First the absolute spectral responsivity of DUT1 and DUT2 are shown graphically in Figs. 6 and 7. The absolute responsivity, determined from the spectral responsivity at wavelengths corresponding to the laser-based measurements, was calculated by linear interpolation. Second, the results from all four measurement systems are summarized in Table 1. The maximum difference among the responsivity values acquired with the four measurement systems is stated with a single number (percentage) calculated from

$$\Delta {\left(\lambda \right)}_{\max}=\frac{\left(R{\left(\lambda \right)}_{\text{hi}}-R{\left(\lambda \right)}_{\text{lo}}\right)}{R{\left(\lambda \right)}_{\text{hi}}}100$$ (2)

where *R*(*λ*)_hi_ is the maximum responsivity value and *R*(*λ*)_lo_ is the minimum responsivity value at wavelength *λ*.

## 8. Discussion

We expect that the responsivity values obtained from services having the lowest uncertainty to be bracketed within the range of values from the other services having a larger uncertainty. In every instance, we find that that our expectation is met. Therefore one can say that the measurements agree to within the stated uncertainty.

The results at 1549.6 nm show the greatest maximum difference between the spectral-responsivity measurement system and the OFPM measurement system. In this case, the difference is approximately 1 %. The reason for this is difficult to know. One possible explanation is based on the fact that DUT2 may have a field of view narrower than is necessary to completely capture light diverging from the end of the fiber connector at longer wavelengths (for example, near 1550 nm). The coupling efficiency and spatial uniformity are both wavelength dependent. It is possible that the coupling efficiency is low for the divergence at 1550 nm, but not at shorter wavelengths (for single mode fiber, the divergence increases with wavelength [14]). The properties of DUT2 have been evaluated, but the divergence for the 1550 nm laser and fiber used for this measurement has not. With knowledge that the coupling efficiency decreases with increasing divergence, we expect *a priori* the fiber-coupled responsivity to be less than the free-field responsivity. We find just the opposite. The fiber coupled responsivity is greater. Other possible explanations are related to the detector’s temperature dependence and the relatively wide bandwidth of the monochromator compared to laser sources. The 1550 nm wavelength is sufficiently shorter than the wavelength of the photodetector’s band edge (which is near 1650) so that we suspect neither temperature biasing, nor the weighting errors that are introduced by the monochromator’s 6 nm bandpass. To make any statements beyond speculation will require further investigation.

## 9. Conclusion and Future Work

The work described in this paper is important in several ways. Since its inception in 1968, there has been only one documented intramural comparison of the C-series laser calorimeter with another primary laser-power measurement standard [15]. This is the first intramural comparison of our own check standards against all four measurement systems that are the basis of national traceability for OFPM calibrations. We have found that measurement results from the various services agree to within our stated uncertainties and thus we have further support that our stated uncertainties are reasonable.

In the coming months, another pair of optical-trap detectors (one based on two temperature-controlled germanium photodiodes [2], the other based on six Si photodiodes [16]) will also be designated as intramural comparison standards. Thus, two pairs of detectors will be available for measurements during the course of a year: one pair will be evaluated during the first half of the year, the other pair for the last half of the year. In time we will develop a history of the detectors while providing a basis for evaluation and recalibration of the various components of the measurement systems.

## Figures and Tables

**Table 1:** Summary of absolute responsivity values (units are A/W, unless stated)

Measurement system^a^	Approximate power level	Relative uncertainty (%, *k* = 2)	DUT1						DUT2		
						Nominal wavelength (nm)					
			514	632.8	672.4	851.5	859.4	1064	1306.5	1549.6	1550.4
SR	10 µW	1.24	0.4055	0.5013	0.5332	0.6763	0.6829	0.2113	0.8877	.9988	0.9990
C4-1	1 mW	0.8		0.5032			0.6848	0.2116			
C4-4				0.5037			0.6849	0.2114			
OFPM	100 µW	0.4			0.5379	0.6822			0.8883	1.0080	
LOCR	1 mW	0.05	0.4046	0.5014				0.2106			1.0062
Maximum difference [Eq. (2)]			0.22 %	0.48 %	0.87 %	0.87 %	0.29 %	0.47 %	0.07 %	0.91 %	0.72 %

a SR = Spectral responsivity measurement system (6 nm bandwidth), C4-1 = C calorimeter, C4-4 = C calorimeter, OFPM = Optical fiber power (fiber coupled), LOCR = Laser optimized cryogenic radiometer.
